# Throwing volume in handball: differences across playing positions and training exercises?

**DOI:** 10.3389/fspor.2026.1824439

**Published:** 2026-07-07

**Authors:** Linda van Maanen-Coppens, Roland van den Tillaar

**Affiliations:** Nord University, Department of Sport Science and Physical Education, Levanger, Norway

**Keywords:** handball, positional differences, shoulder injuries, throwing volume, training exercises

## Abstract

**Introduction:**

Despite high prevalence of shoulder injuries in handball, little is known about the specific throwing loads that may contribute to injury risk. Therefore, this exploratory study aimed to quantify throwing volume in young elite handball players during regular training and to examine differences across playing positions and exercise types.

**Methods:**

Sixteen healthy elite youth female handball players were monitored during four regular training sessions (381 minutes total). Training sessions were video recorded from three camera angles, and all throws were manually tagged per player as passes or shots. Training content was categorized into eight exercise types, and throwing frequency was calculated as throws per minute based on active exercise time.

**Results:**

Back players significantly throw more than wing or pivot players (175.9±60.1 vs 85.4±23 vs 105.8±22.8, p<0.001, *η*²≥0.45). Furthermore, throwing volume was strongly influenced by the type of training exercise (p<0.001, *η*²≥0.27). Highest passing volume was seen during passing exercises for all playing positions (back 38.5±13.9, wing 37±12.4, pivot 38±4.7), and most shots were performed during goalkeeper warming up (back 7.5±1.2, wing 7.7±0.9, pivot 7.5±1.3). Additionally, the number of both passes and shots was high during small-sided game exercises on half field with equal to or more than 4x4 players, with large positional differences.

**Discussion:**

Throwing volume in elite youth handball players varies substantially by playing position and training exercise. These findings highlight the importance of considering both positional demands and exercise structure when designing training programs, with potential implications for load management and injury prevention.

## 1 Introduction

Handball is a popular sport that has been recognized as one of the Olympic sports with the highest injury prevalence (1). The sport is characterized by fast pace defensive and offensive actions like throwing during the game, with the objective of scoring goals. It is known for its complexity with high level of physical demand and variations, combined with technical and tactical aspects. Shooting in handball is associated with large shoulder abduction and external shoulder rotation (2). As a result, the shoulder is the most affected region for upper extremity injuries in handball, accounting for 4%–27% of all injuries (3, 4) and 41%–57% of the players experience shoulder pain during their handball career. Thereby, the impact is high as Norwegian studies showed that 68.3% of the handball players had changed their normal training load because of shoulder pain. During matches, 52.3% of the players reported pain. Furthermore, players also reported pain during their daily life activities (4, 5).

The current literature mostly focuses on intrinsic risk factors and prevention strategies for shoulder injuries, such as reducing imbalances in strength or range of motion. Monitoring parameters are not used to identify athletes at risk for obtaining shoulder injury. Earlier studies focus on number of matches or training hours, but specific data about throwing volume in handball is currently lacking.

The amount of training hours was reported to be an injury-risk-determining factor for injuries (6–8). To improve performance, a certain threshold needs to be exceeded to stimulate adaptation. However, when the amount of overload becomes excessive, this can lead to unfunctional overreaching and the athlete might not be able to recover. These possible negative outcomes increase the risk of injuries (9, 10).

It is reported that elite handball players perform approximately 48.000 throwing movements per season with ball velocities that can reach up to 130 km/h (4). Additionally, handball throws are performed with a great variety of intensity and technical execution (11–14). According to Prestkvern (15), elite handball players complete 1,200 throws per training week but large differences between playing positions may occur, both in training and matches. Back players seem to pass and shoot more often compared to their teammates, which might increase their risk for shoulder injuries (13, 16). However, earlier studies have mostly investigated match data, while this is just a small part of the total throwing volume per week. More insight into the number of throws during training will provide valuable data for future adaptations to training loads and preventing shoulder injuries. Additionally, individual training prescriptions should be optimized by monitoring the training load carefully. To the best of our knowledge, no studies have been done to determine and compare throwing volume in several types of training exercises.

Therefore, the main aim of the study was to investigate the throwing volume (absolute number and frequency of passes and shots) in young elite team handball players during regular training, and secondly to study if there are differences in throwing volume between playing position and training exercises during training. It was hypothesized that, as shoulder complaints seem to be more common in back players compared to wing or line players, this is probably due to a higher throwing load during training (16). It is therefore expected that in this study, back players will show a higher throwing volume compared to other positions. Furthermore, it is expected that during handball training, some training exercises would consist of a higher throwing volume than other exercises, which could be important information for planning throwing volume load.

## 2 Material and methods

### 2.1 Participants

For this explorative study, sixteen young female handball players (age=16.9 ± 1.3) were included (seven left-handed, nine right-handed players). Goalkeepers were excluded from the study. All players were recruited from the Handball Academy in the Netherlands; a full-time multi-year training program for the most promising Dutch handball talents, preparing them for (inter)national elite competitions in the future, thereby classified as tier 4 of their age category. Their training schedule includes approximately 8–10 h of ball training and 5–7 h of strength and conditioning training per week. All players were part of national youth selections and competed at the highest national and international level for their age group. All players were healthy, physically fit, and injury-free at the time of the study. All players voluntarily participated in the study. Informed consent was obtained from all participants and, for those under 18 years of age, from their parents/guardians. The study was approved by the Norwegian Agency for Shared Services in Education and Research (project number: 393779) following the most recent revision of the Helsinki Declaration.

### 2.2 Procedures

In July 2025, four regular ball training sessions were observed and analyzed during this study, including 381 min of training in total. The training groups were based on age categories: two training sessions for the age group under seventeen years (187 min) and two training sessions for the age group under nineteen years (194 min). Training sessions were performed on different days, with a 24-hour rest period in between. During each training session, seven players in different playing positions were monitored; four back players, two wing players and one pivot per group per session. All training sessions were recorded from three different camera positions, to capture all players and throws from different angles; two cameras were mounted on the ceiling, each focused on one half of the court. A third camera was positioned on the sideline, at the middle of the court, to follow the players.

### 2.3 Data analysis

All four training sessions were regular training sessions that handball players would have during the week and consisted of exercises that are considered normal for handball practice. After each training session, the recorded videos were watched multiple times by the researcher, and all throws were tagged and labeled per individual player. Different types of throwing were distinguished as passes or shots. Absolute numbers of total throws, passes and shots per player per session were calculated. During training different training exercises were divided into eight different types of training exercises:

- Warming up (including warm-up games)
- Passing exercises
- Goalkeeper warming up, in which players line up and shoot at the goalkeepers
- Individual development plan exercises (IOP), in which players choose which type of shooting/technique they want to improve in this exercise, also including positional shots
- Small-sided games on half field, with less than 4vs4 players
- Small-sided games on half field, with equal to or more than 4vs4 players
- Small-sided games on whole field, with less than 4vs4 players
- Small-sided games on whole field, with equal to or more than 4vs4 players

For each training exercise, the active training time was calculated from the first throw until the last throw in that specific phase of the training. Based on the active training time, the throwing frequency in throws per minute was calculated by dividing the number of throws by the time for each specific training exercise and per position.

### 2.4 Statistical analysis

Median and range were calculated for descriptive characteristics about the participating players, as well as for the absolute number of throws, passes and shots, due to the low number of players in each position. For the throwing types and training exercises, descriptive statistics were calculated in means, standard deviations, and 95% confidence intervals. The Shapiro–Wilk test was used to confirm normality of variables. When data was not normally distributed non-parametric Kruskall-Wallis and Friedman tests were conducted as non-parametric tests. Analyses of variance (ANOVA) with repeated measures were used to investigate the number of throws, passes and shots during training and frequency of passes and shots (number per minute) in relation to playing position and training exercise. When sphericity was violated, Greenhouse-Geisser corrections were applied.

For all statistical procedures, a *p*-value of *p* < 0.05 is used as a cut-off for significance. When significant effects were found, Holm-Bonferroni *post hoc* tests were used to identify differences between playing positions or training exercises. Effect size was evaluated with (Eta partial squared) where 0.01<*η*^2^ < 0.06 constitutes a small effect, a medium effect when 0.06<*η*^2^ < 0.14 and a large effect when *η*^2^ > 0.14 (17). All statistical analyses were conducted using version 0.95.4. of JASP (University of Amsterdam, Amsterdam, Netherlands).

## 3 Results

### 3.1 Total number of passes and shots

Four training sessions were recorded and analyzed in the study, with sixteen players (seven left-handed, nine right-handed players) in total. During each training session, seven players in different playing positions were monitored. Twelve players participated in two training sessions, and four players only participated in one training session. This resulted in 28 individual measurements. In total, 3,921 throws were recorded and analyzed over the four training sessions.

**Figure 1:**
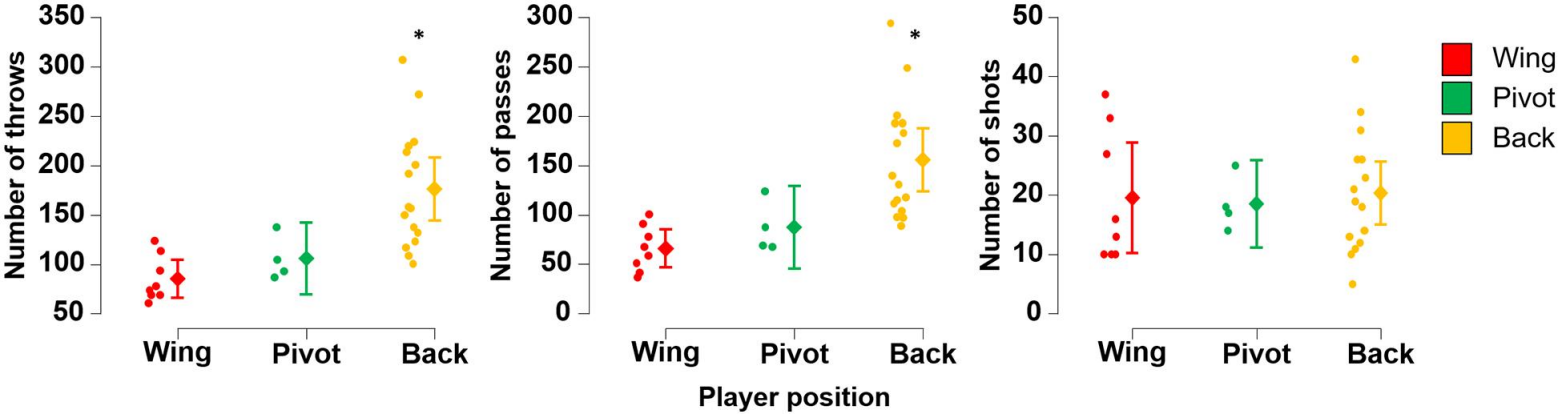
Means (±95% CI error bars) for the total number of throws per player position, the total number of passes per player position, and the total number of shots per player position. * indicates a significant difference between back with wing players.

The average number of total throws per player was 123.5 per training session (range 61–307). When the total number of throws was divided into passes and shots, the average number of passes per player was 102.5 (range 37–294) and for shots the average number per player was 18 (range 5–43). Significant differences in the average number of passes and of total throws between playing positions were found (F ≥ 10.14, *p* < 0.001, *η*^2^ ≥ 0.45, Figure 1), while no significant difference in average number of shots between playing positions were found (F = 0.06, *p* < 0.942, *η*^2^ < 0.01, Figure 1). Holm-Bonferroni *post hoc* tests showed that back players had significantly more throws and passes than wing players (*p* < 0.001), while no other significant differences between positions were found.

### 3.2 Differences in training exercises

**Figure 2:**
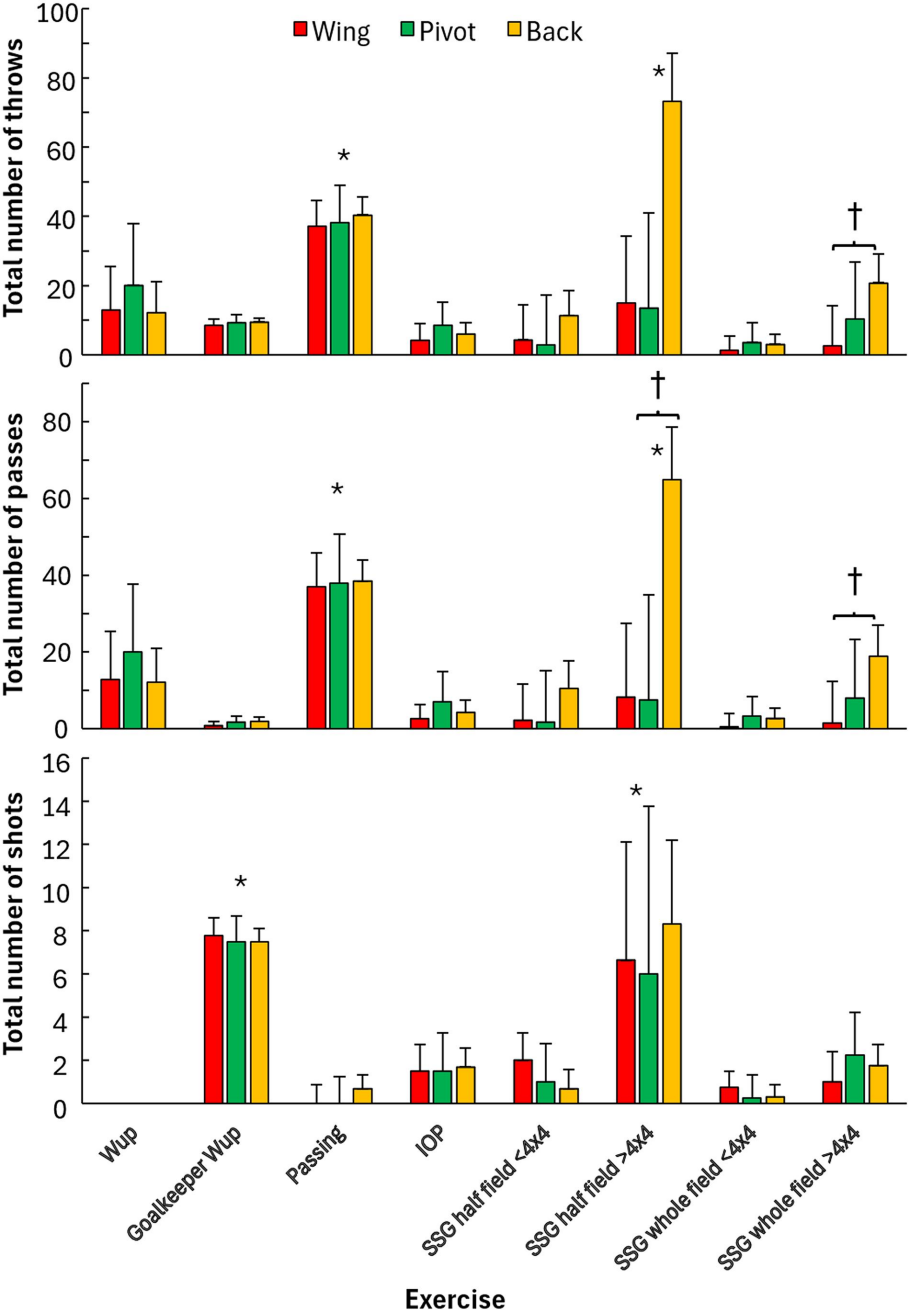
Means (95% CI) for total number of throws, passes and shots per exercise per position, * indicates a significant difference with all other exercises except not with the other *. † indicates a significant difference with all other positions for this exercise.

The total number of throws and passes differed significantly between exercises (F ≥ 19.1, *p* < 0.001, *η*^2^ ≥ 0.27). Holm-Bonferroni *post-hoc* comparison showed that the number of total throws and passes was significantly higher during the passing exercises compared to almost all other training exercises, except for the small-sided game exercises on half field with equal to or more than 4 × 4 players. Large positional differences were seen in the small-sided game formats, where back players consistently had more passes and total throws than the wing and pivot players in both half- and whole-field formats (Figure 2).

The variation in the number shots (F = 22.6, *p* < 0.001, *η*^2^ = 0.44) across different training exercises was even larger than it was for the total number of throws or passes (Figure 2). Holm-Bonferroni *post-hoc* comparison revealed that almost no shots were performed during warming up, passing exercises and the small-sided game exercise with less than 4 × 4 players on the field. The number of shots were significantly higher compared to other training exercises during the goalkeeper warming up and the small-sided games exercises on half field with equal to or more than 4 × 4 players. Unlike total throws and passing, player position did not significantly influence the number of shots (Figure 2).

### 3.3 Frequency of passes and shots (number per minute)

During this study, 381 min of training were analyzed and divided into different training phases based on the type of training exercise. The average throwing frequency for all throws was 1.69 throws per minute (range 0.85–4.68). Players performed about 1.46 passes per minute (range 0.46–4.49) and 0.22 shots per minute (range 0.07–0.53). A significant difference was seen in the overall throwing frequency (F = 8.62, *p* = 0.001, *η*^2^ = 0.18), where back players had a higher throwing frequency compared to wing players (*p* = 0.002).

**Figure 3:**
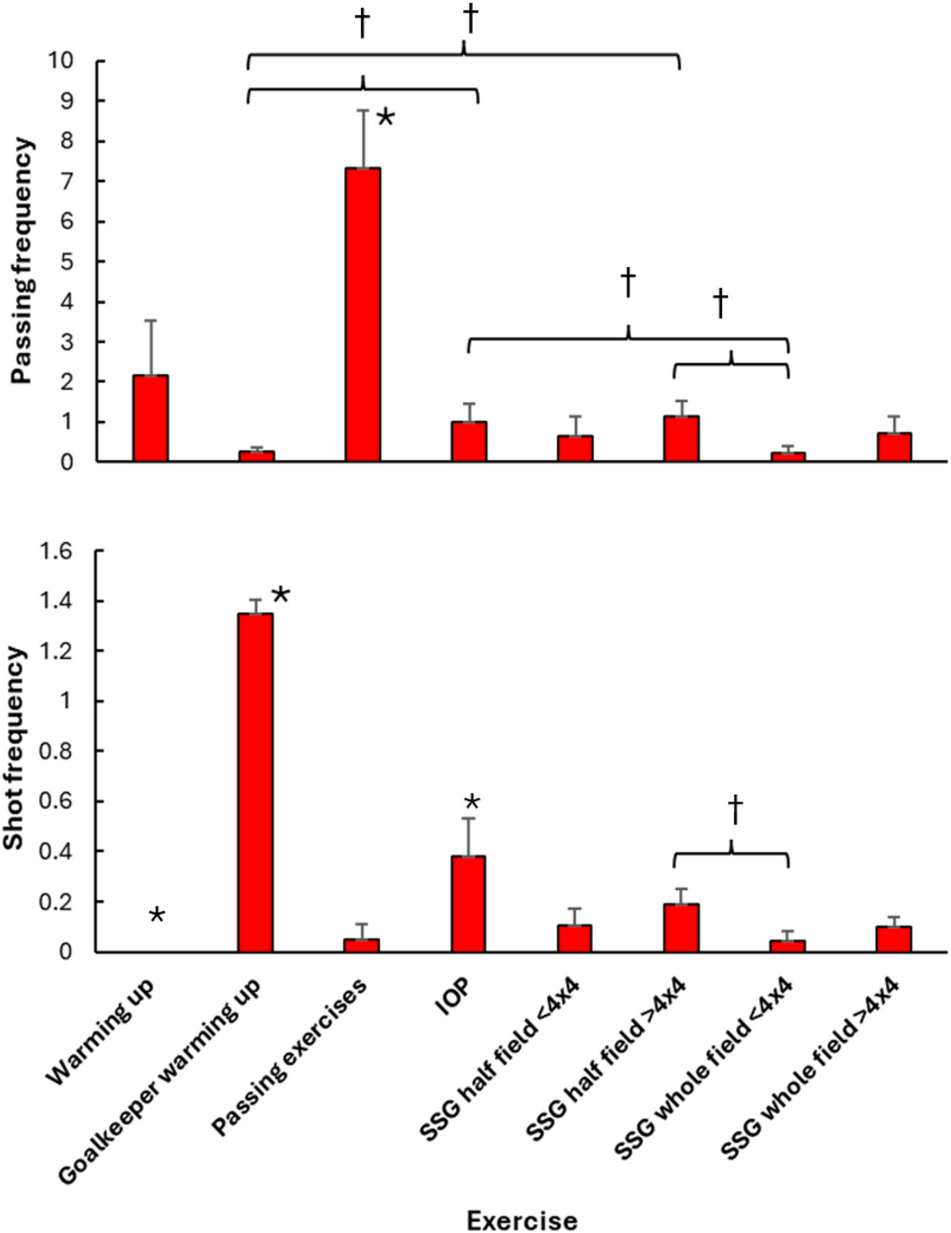
Means (95% CI) for passing and shot frequency per exercise. * indicates a significant difference with all other exercises. † indicates a significant difference between these two exercises.

The effect of training exercise on the passing frequency (Figure 3) was significant and large (F = 43.68, *p* < 0.001, *η*^2^ = 0.62). Highest passing frequency was shown during passing exercises (M = 7.32 ± 3.73), and lowest passing frequency was shown during small-sided games on whole field with equal to or more than 4 × 4 players (M = 0.22 ± 0.5).

The shot frequency (Figure 3) also strongly differed across training exercises (F = 176.00, *p* < 0.001, $\eta^{2}$ = 0.867. The shot frequency was highest during goalkeeper warming up, exceeding all other exercises (*p* ≤ 0.001). Additionally, the shot frequency during individual development plan exercises was significantly higher than during most other exercises. During warming up and passing exercises, the shot frequency was nearly zero.

## 4 Discussion

The aim of this explorative study was to gather information about the number and frequency of throws in young elite team handball players. The present study examined positional and activity-specific differences in total throws, passes and shots during training.

During this study, the average number of total throws per player was 123.5 (range 61–307) per training session. This may seem slightly low compared to the average 120–150 throws as found in previous studies, but it should be noted that only two sessions per training group were included in the current study and substantial individual differences were seen (18, 19). To get a complete picture of the total throwing load, it would be beneficial to follow players for a longer period. Ideally, the entire season should be evaluated to identify possible differences per period due to seasonal periodization. However, like previous studies, the total number of throws was strongly dependent on the playing position. Back players showed the largest volumes of throws and passes, especially in the small-sided game formats. This confirms results from previous studies from Karcher and Saal et al. (13, 20), where back players showed significantly more passes than wing and pivot players. The total number of throws or passes may seem different in the current research; however, this might be related to the fact that both the studies from Karcher and Saal et al. (13, 20) were based on match data while the current study used training data.

The fact that back players seem to have a consistently higher load also has consequences for the risk of overload, especially of the shoulder. Previous studies have identified that incorrect dosing of the total handball load is a risk factor for developing shoulder injuries in handball players, (21, 22). The position-specific high load thus increases the need for targeted monitoring and preventive measures for back players.

In contrast to an earlier study from Pueo, Tortosa-Martínez (23), based on match data, no significant differences were found between player positions for the number of shots. A possible explanation for this could be the limited number of players and training sessions within this study. All training sessions did include exercises where the assignment was the same for each player position when it comes to shooting the ball at the goal, for example during goalkeeper warming up. The highest shot frequency was seen during the goalkeeper warming up, which means that players perform many shots in a short time frame, often with a high intensity. This is important information for coaches and medical staff when it comes to load management, especially in case of injury prevention or return to play after shoulder injuries.

Overall, the total number of throws was highly dependent on the type of exercise. During passing exercises, a high number of throws were seen across the entire group, accompanied by a high passing frequency. In contrast, large positional differences were seen during the small-sided games, with mainly more throws for the back players. Reviews of small-sided games emphasize that altering the playing field or number of players can redistribute contact and finishing opportunities, matching this pattern (24, 25). Handball trainers can use this information to choose the training exercises that suit their periodization goal: choose passing drills when the aim is high-repetition exposure across the total squad; and use half-field small-sided games to emphasize decision-making under pressure with elevated distribution loads for back players.

A clear limitation for this study was the limited number of participants and training sessions. To get a complete understanding of the throwing volume during a handball season and its possible influence on shoulder injuries, more extensive research including more training sessions and participants from each position is needed. However, the methods used for the current study make it rather difficult and very time-consuming. In addition, it should be mentioned that the study provides a detailed count of throws but does not account for the intensity or quality of these actions. A pass in a low-intensity warm-up drill does not provide the same physiological or mechanical load as a maximal-effort jump shot during a small-sided game. With this knowledge, it might be of interest to further examine the physical load on the upper extremities during throwing in handball. Moreover, it is relevant to investigate how much handball throwing load players can tolerate while enhancing throwing performance, without developing injuries. Monitoring the throwing volume with inertial measurement units (IMUs), instead of manually tagging all throws using video recordings, might be a potential solution (18, 26).

## 5 Conclusion

Results of this study show that the throwing volume in handball was strongly influenced by the type of training exercise, with possible implications for load management and injury prevention. The influence of playing position varies depending on the type of throw (passes vs. shots). Most throws were performed during passing exercises, goalkeeper warming up and small-sided game exercises on half field with equal to or more than 4 × 4 players. In general, back players show the highest throwing load. Further research is needed to assess and periodize the throwing volume during the season, to optimize performance while limiting overuse risk.

## Data Availability

The raw data supporting the conclusions of this article will be made available by the authors, without undue reservation.
